# The Effect of Interleukin 38 on Angiogenesis in a Model of Oxygen-induced Retinopathy

**DOI:** 10.1038/s41598-017-03079-z

**Published:** 2017-06-05

**Authors:** Jing Zhang, Ruijuan Zhao, Jianping Chen, Jiayi Jin, Ying Yu, Yunzhe Tian, Weihua Li, Wencong Wang, Hongyan Zhou, Shao Bo Su

**Affiliations:** State Key Laboratory of Ophthalmology, Zhongshan Ophthalmic Center, Sun Yat-sen University, Guangzhou, 510060 China

## Abstract

Interleukin 38 (IL-38) is a novel identified cytokine of IL-1 family in which some members are important in inflammation and angiogenesis. However, the role of IL-38 in regulating angiogenesis is unknown. The aim of the present study is to explore the effect of IL-38 on angiogenesis. Oxygen-induced retinopathy (OIR) of C57BL/6 J mice was induced by exposure of hyperoxia (75% oxygen) from postnatal day 7 (P7) to P12 and then returned to room air. The mice were injected with IL-38. At P17, neovascular region (tufts) and avascular area of the retinas were analyzed. The data showed that administration of IL-38 *in vivo* inhibited retinal angiogenesis significantly. Furthermore, the addition of IL-38 to the cell cultures attenuated the proliferation, scratch wound healing and tube formation of vascular endothelial cells induced by VEGF significantly. Our findings suggest that IL-38 is an antiangiogenic cytokine in pathophysiological settings and may have therapeutic potential for angiogenesis related diseases.

## Introduction

Angiogenesis is a process of new blood vessels sprouting from pre-existing vasculature, which has an important role in reproduction, organ regeneration and wound repair^1, 2^. Pathological angiogenic signaling results in disordered neovascularization and persist for years, such as retinopathy of prematurity (ROP), age-related macular degeneration (AMD), corneal neovascularization (CNV), tumor formation, rheumatoid arthritis and atherosclerosis. Angiogenesis is tightly orchestrated by a range of angiogenic cytokines such as vascular endothelial growth factor (VEGF), basic fibroblast growth factor (bFGF), transforming growth factor β (TGF-β) and interleukin 1β (IL-1β)^3^ and antiangiogenic cytokines. These cytokines act on the process of proliferation and migration of endothelial cells (ECs) which is regarded as the hallmark of angiogenesis^4, 5^. The current intervention of modulating the prominent angiogenic cytokines such as anti-VEGF therapy has shown efficacy in ocular neovascularisation^6^. However, a proportion of patients are refractory to anti-VEGF agents, suggesting other angiogenic or antiangiogenic cytokines that coordinately contribute to angiogenesis remain to be identified^7^.

Interleukin 38 (IL-38, also named IL-1F10, IL1HY2, FIL-1θ, or IL-1θ) is a novel member of interleukin 1 family. Human IL-38 encodes a 152 amino acid protein with 16.9 kD molecular mass^8, 9^. It has been detected in a range of tissues including heart, placenta, fetal liver, skin, spleen, thymus and tonsil by multitissue first-strand cDNA PCR analysis^8^. Human IL-38 gene is located in the IL-1 family cluster on chromosome 2q13-14.1, between the genes encoding IL-36Ra and IL-1Ra^10^. IL-38 gene polymorphisms are associated with psoriatic arthritis (PsA), ankylosing spondylitis (AS)^11–13^ and cardiovascular disease^14^. As is typical of the IL-1 family, including IL-36Ra, IL-36*α*, IL-36*β* and IL-36*γ*, IL-38 lacks a signal peptide and caspase 1 consensus cleavage site^8, 15^. However, the natural N terminus for IL-38 is still unclear^15^. In addition, similar to the crystal structure of IL-1Ra and IL-1^8, 16^, IL-38 displays a 12-*β*-stranded trefoil structure and shares. These features of IL-38 further prove that it belongs to the IL-1 family.

IL-1 family molecules play a prominent role in inflammatory and immune responses, acting as the first line of defense against invasive pathogenic microorganisms and physical damage. However, many cytokines of the IL-1 family, such as IL-1α, IL-1β, IL-18, IL-33 and IL-37 contribute importantly to angiogenesis^7, 17–19^. IL-38 shares high sequence homology (41% and 43%) with IL-1Ra and IL-36Ra^9, 15^ and lower homology (14–30%) with IL-1*β* and other IL-1 family proteins. IL-38 was recently suggested to bind to IL-36R and exerted antagonistic effects similar to those of IL-36Ra^20^. In a report by van de Veerdonk, IL-38 bound to IL-36R but did not bind to the immobilized IL-1RI, IL-18R and IL-1R accessory proteins (IL-1RAP, IL-1RAcP)^20^. However, IL-1RI was once considered a receptor for IL-38^8, 20^, although with lower affinity than IL-1 or IL-1Ra^8^. The issue about receptor of IL-38 is still in dispute. The studies suggest that IL-38 may act as an IL-1 family antagonist (likely belongs to IL-36 subfamily) exerting its function and is probably involved in angiogenesis-associated pathophysiologies. For example, a recent study showed that IL-38 was upregulated in the serum of patients with systemic lupus erythematosus (SLE), probably exerting antiinflammatory functions^21^. Moreover, IL-38 is associated with pathogenesis of spondyloarthritis^22, 23^ and is upregulated in the salivary glands of patients with primary Sjoren’s syndrome^24^. To date, no reports have described the effect of IL-38 on angiogenesis.

In this study, we found that administration of IL-38 *in vivo* inhibited retinal angiogenesis significantly. IL-38 attenuated the proliferation, migration, and tube formation of endothelial cells in a dose-dependent manner. The inhibitory effect of IL-38 was eliminated by adding anti-IL-38 antibody. These results suggest that IL-38 is a regulator of pathophysiological angiogenesis.

## Results

### IL-38 Regulates Developmental Angiogenesis in Neonatal Mice

**Figure 1:**
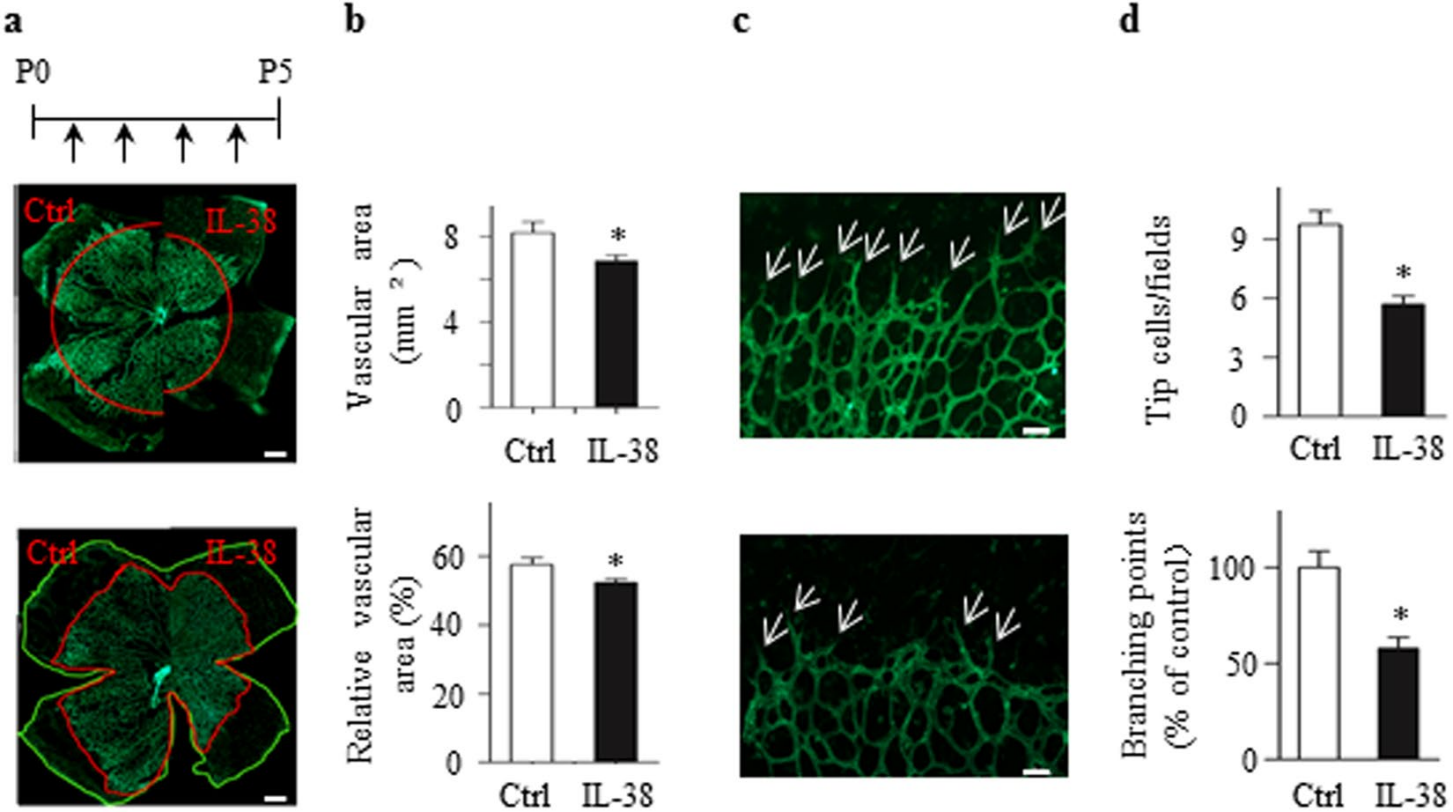
IL-38 inhibits developmental angiogenesis. (**a**) Neonatal mice were intraperitoneally administrated with IL-38 (1 ng per gram bodyweight) from P1 to P4. Vascular area of the retina whole mounts was assessed by fluorescence microscopy. The retina whole mounts and vascular area were respectively delineated by green and red boundary line. Scale bars, 500 μm. (**b**) Vascular area and relative vascular area from control group and IL-38-treated group were summarized; n = 8. (**c**) High-magnification images of retinal vasculature from control mice (top) or IL-38-treated mice (bottom). (**d**) Densities of tip cells (white arrow) and branching points were quantified. Data from 8 animals were summarized in the graphs. Scale bar, 50 μm. Data are presented as mean ± SEM. *P < 0.05 Student *t* test.

We first investigated whether IL-38 regulates neovascularization under physiological conditions. The retinal vascularization is a widely used model for physiological angiogenesis. Neonatal mice were intraperitoneally administrated with IL-38 (1 ng/g body weight) from postnatal day 1 (P1) to P4 (Fig. 1a). The mice were euthanatized at P5 and the retinas were harvested, fixed and flat-mounted for isolectin B4 (IB4) staining (Fig. 1a). After IL-38 administration, the vascular area and relative vascular area were significantly attenuated compared to the control group (Fig. 1b). The vessel density and tip cell numbers observed under immunofluorescence microscopy were also decreased in IL-38-treated mice (Fig. 1c and d).

### IL-38 Inhibits Pathological Angiogenesis

**Figure 2:**
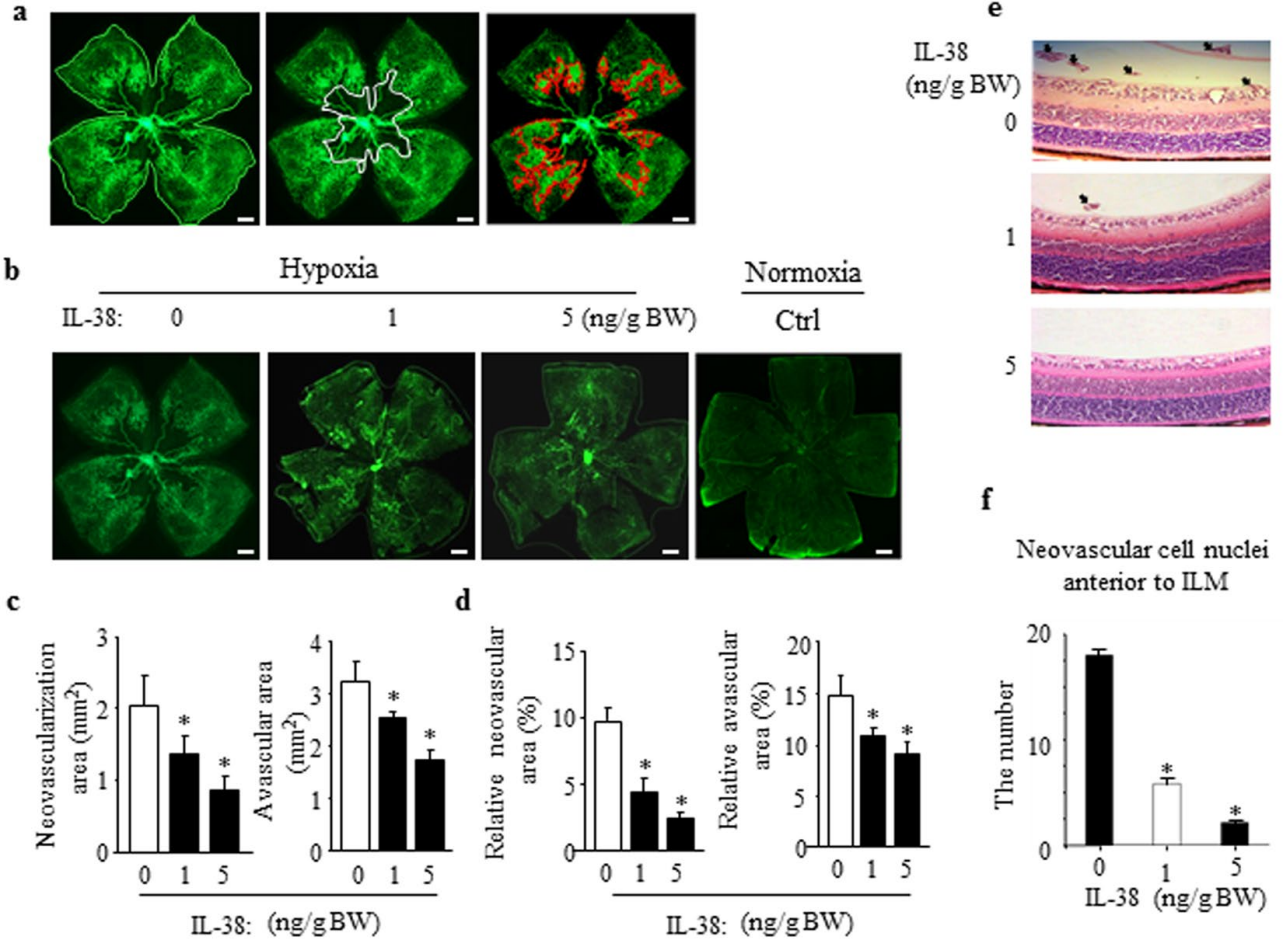
Systemic administration of IL-38 inhibits pathological angiogenesis in mouse OIR model. (**a**) Neovascular region, avascular region and the retina whole mounts delineated respectively by red, white and green boundary line. Representative images from the vehicle (OIR mouse model) are shown. (**b**) Mice were administrated with PBS, 1 or 5 ng/g bodyweight of IL-38 at P12, P14, and P16 during normoxia phase. (**c**) Disordered neovascular regions and avascular regions were quantified. (**d**) The relative retinal neovascular and avascular areas were quantified. (**e**) Neovascular cell nuclei anterior to internal limiting membrane (ILM) represented extent of retinal neovascularization and are indicated with black arrows in retina. (**f**) Statistical analysis showed that the number of neovascular cell nuclei anterior to the ILM in the IL-38-treated group is significantly decreased compared with the counterpart mice at P17. Scale bars, 500 μm. n = 8 per group. *P < 0.05 Student *t* test. BW, bodyweight.

In the mouse model of oxygen-induced retinopathy (OIR), hypoxia-triggered angiogenic response induces the formation of pathological neovessels and neovascularization tufts are considered as the hallmark for characterization of pathological angiogenesis. The retinas of OIR mice at P17 were harvested, fixed and flat-mounted for IB4 staining. As is shown in Fig. 2a, the retinas of the mice developed extensive vascular regression in the central region and pathologic neovascular tufts in the middle and peripheral regions. The avascualar area and neovascular tufts in the groups with IL-38 intraperitoneal admistration (1 or 5 ng/g body weight) were reduced significantly, as compared with that of control group (Fig. 2b–d). Figure 2e is the representative, illustrating neovascular cell nuclei anterior to the internal limiting membrane (ILM). Consistent with IB4 staining analysis, the number of neovascular cell nuclei in the IL-38-treated group was significantly decreased compared with the counterpart control (Fig. 2f).

**Figure 3:**
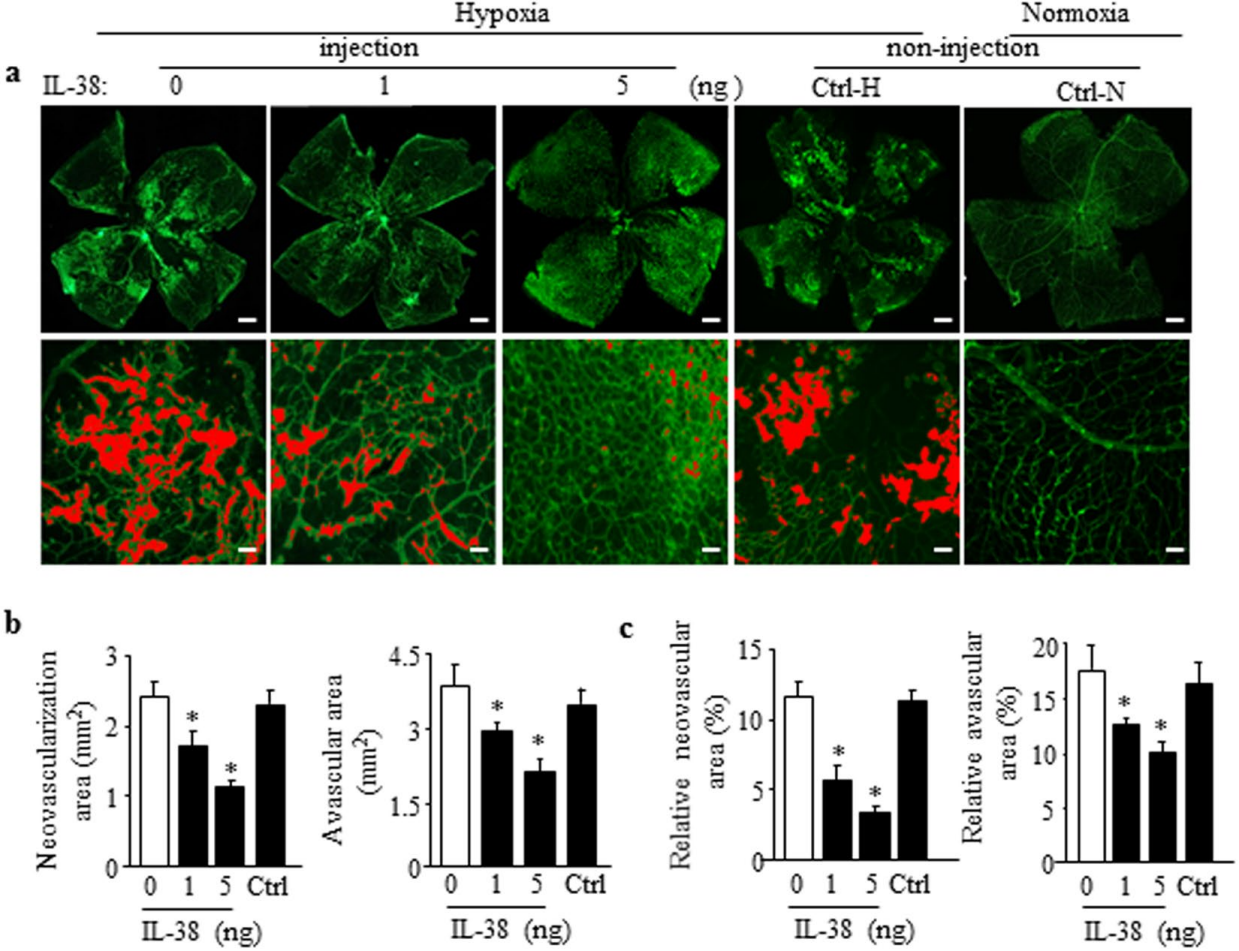
Local administration of IL-38 inhibits pathological angiogenesis in mouse OIR model. (**a**) Mouse retinas were harvested at P17 and subjected to whole mount immunostaining with isolectin B4. Mice were injected inravitreally with 1 μm l IL-38 dissolved in PBS at different concentrations (1 or 5 ng/μl) at P12, during normoxia phase. Disordered neovascular growth (tufts; lower) in retina were highlighted in red and quantified. (**b**) The retinal neovascular and avascular areas were quantified. (**c**) The relative retinal neovascular and avascular areas were quantified. Scale bars, 500 μm (a, upper) and 100 μm (a, lower). n = 8 per group. *P < 0.05 Student *t* test.

In addition, the neovascularization tufts of the OIR mice administrated intravitreally with IL-38 (1 or 5 ng) at P12, was significantly decreased in a dose-dependent manner by IB4-isolectin staining imaging analysis at P17 (Fig. 3a). The sizes of vascular and avascular region were markedly reduced compared with the vehicle treated group (Fig. 3b and c).

### IL-38 Attenuates Endothelial Cell Proliferation

**Figure 4:**
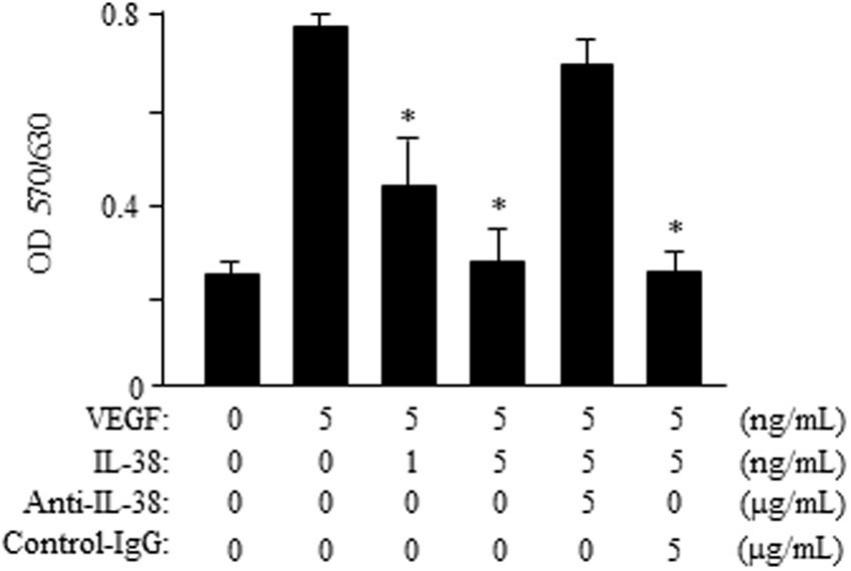
IL-38 attenuates endothelial cell proliferation. HRECs (1 × 10^4^) were cultured with VEGF/IL-38/anti-IL-38/IgG at 37 °C in a CO_2_ incubator and the proliferation was determined by MTT method. Shown are results from one representative experiment of three performed (in triplicates). *P < 0.05 Student *t* test.

The hallmark of angiogenesis is the proliferation and migration of endothelial cells^5^. We examined the effects of IL-38 on proliferation of cultured human retinal endothelial cells (HRECs) and human umbilical vein endothelial cells (HUVECs). We found that supplementation of IL-38 in cell culture significantly reduced VEGF-induced HREC proliferation in comparison to the control cells at concentration of 1 or 5 ng/mL (Fig. 4). The inhibitory effect of IL-38 on HRECs was eliminated by adding anti-IL-38 antibodies (Fig. 4). Similar to HRECs, addition of IL-38 to the culture reduced the proliferation of HUVECs (Supplementary Figure 3). The results demonstrate the inhibitory effect of IL-38 on ECs proliferation.

### Effects of IL-38 on Endothelial Cell Migration

**Figure 5:**
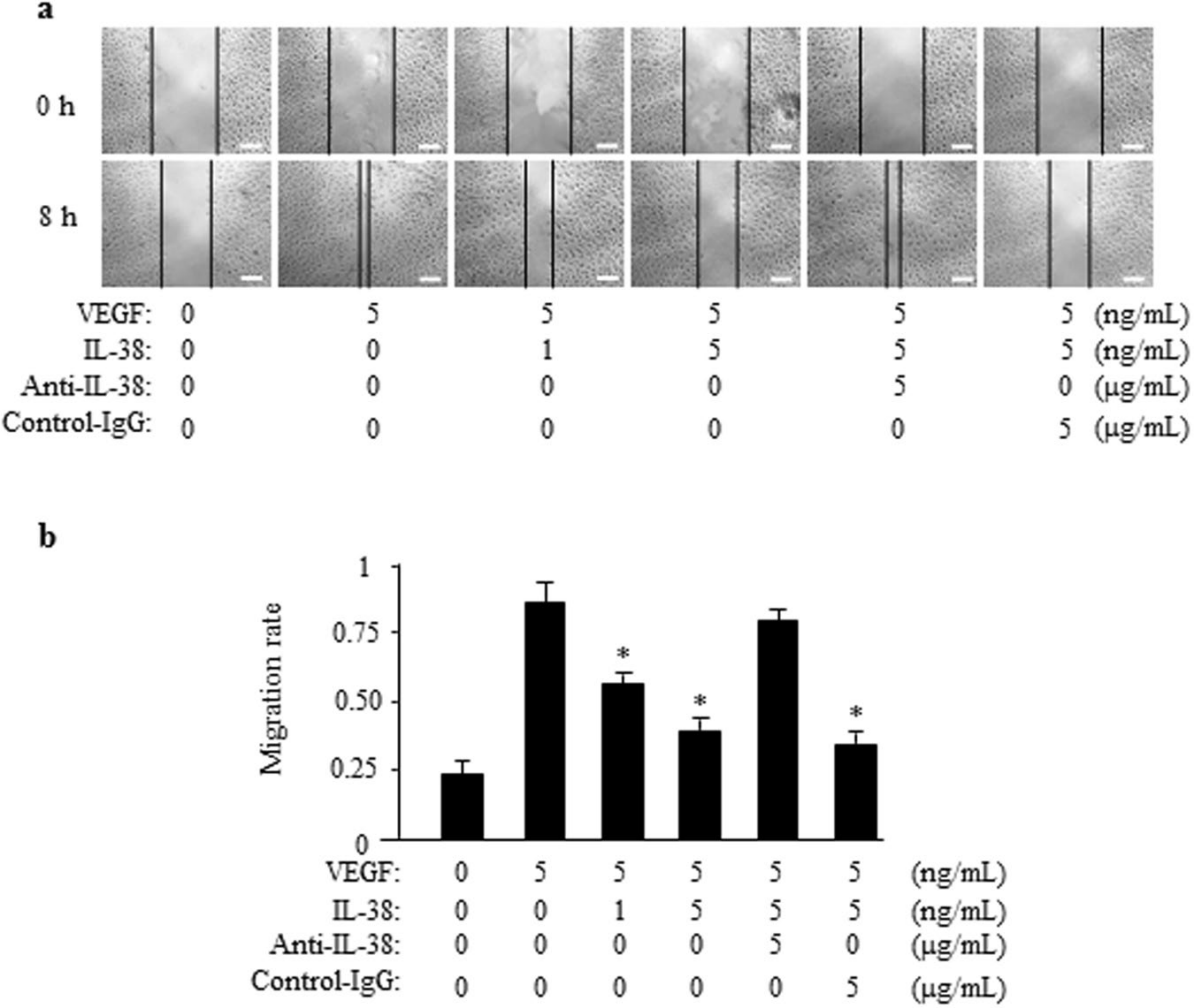
IL-38 reduces endothelial cell migration. (**a**) The effect of IL-38 on HRECs migration in scratch wound assay. Cells were treated with indicated concentrations of IL-38. Representative images after 0 and 8 hours after scratch wounding were shown. Scale bars, 100 μm. (**b**) Quantification of IL-38-treated HRECs migration in monolayer scratch wound assay. *P < 0.05 Student *t* test.

Migration of ECs toward the angiogenic stimulus is an important early process in angiogenesis^25^. We next determined the effect of IL-38 on EC migration. After a scratch wound was created on the monolayer of HRECs, 1 or 5 ng/mL of IL-38 were added to the culture and the cells were monitored 0 and 8 hours (Fig. 5a). The healing area of HRECs in IL-38-treated group was reduced than that of control group. The inhibitory effect of IL-38 was eliminated by adding anti-IL-38 antibodies (Fig. 5). Similar to HRECs, addition of IL-38 to the culture reduced the migration of HUVECs (Supplementary Figure 4).

### IL-38 Suppresses Endothelial Cell Tube Formation

**Figure 6:**
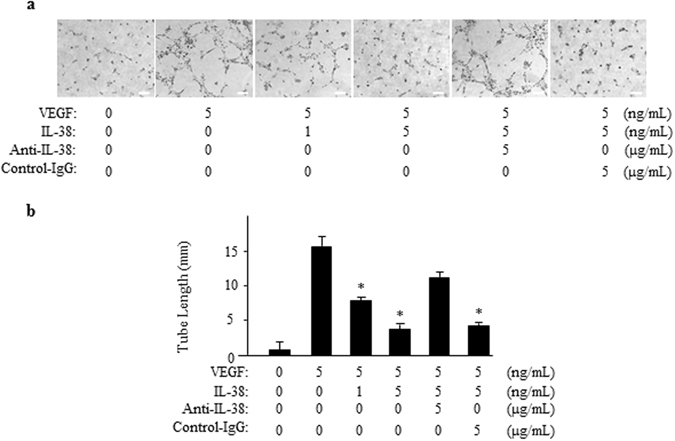
IL-38 reduces endothelial cell tube formation. (**a**) 40,000 HRECs/well were seeded on Matrigel containing VEGF/IL-38/anti-IL-38/IgG in depleted medium. The cells were cultured for 18 h at 37 °C 5% CO_2_. Tube formation was quantified by counting the tube-like structures in the gel and data were presented as the number of branches per field. Scale bar, 200 μm. (**b**) Total length of tubule structure were quantified. *P < 0.05 Student *t* test.

We then performed tube formation assay to identify the *in vitro* anti-angiogenic activity of IL-38. Capillary-like tube formation was induced in VEGF-stimulated HRECs (Fig. 6a). IL-38 effectively inhibited the tube formation in dose-dependent manner as quantified by total tubule length and branching points (Fig. 6b). The inhibitory effect of IL-38 on tuber formation was eliminated by adding anti-IL-38 antibodies. Similar to HRECs, addition of IL-38 to the culture reduced the tube formation of HUVECs (Supplementary Figure 5).

## Discussion

Our results identified IL-38 as an inhibitor in both pathological and physiological settings. IL-38 is the 10th member of IL-1 family and emerging regarded as an important anti-inflammatory cytokine. The most identified IL-1 family members are widely expressed in inflammatory cells and exert important role in inflammatory responses. Recently, the relationship between inflammation and angiogenesis has attracted more attention. Many cytokines of IL-1 family are closely associated with angiogenesis^7, 17–19^. To our knowledge, the role of IL-38 on angiogenesis has not been investigated before. We found that IL-38 was in low expression at P17 in normal mice, but it was up-regulated at P17 in OIR mice (Supplementary Figure 1a,b). IL-38 was highly expressed at P0 but the expression at P7, P12, P15 and P17 was decreased as compared with P0 in normal mice (Supplementary Figure 1c). That is, IL-38 is highly expressed at avascular situation and but decreased after vascular growth. Whether IL-38 is involved in regulating angiogenesis when the retinal vasculature is fully developed need further study.

Retinal neovascularization is a main cause of proliferative diabetic retinopathy (PDR), which can lead to vision loss due to secondary complications such as blood retinal barrier breakdown, vascular leakage, inflammation and edema^26–29^. In this study, we further demonstrated that IL-38 significantly suppressed neovascularization in the mouse model of OIR. Based on our results, local and systemic application of IL-38 had an equivalent effect on inhibiting angiogenesis. These results suggest that IL-38 is an antiangiogenic cytokine that inhibits retinal angiogenesis. In addition, we found that IL-38 expression was increased at P12 in OIR mice but decreased after that (Supplementary Figure 1d). The result indicates that IL-38 expression is increased in hyperoxia situation, a well-known anti-angiogenic conditions that stimulates vascular vaso-obliteration. Furthermore, we have demonstrated the antiangiogenic effect of IL-38 *in vitro* model of the cells. IL-38 attenuates VEGF-induced EC proliferation, migration and tube formation. The inhibitory effect of IL-38 was eliminated by adding anti-IL-38 antibodies. However, the mechanism by which IL-38 inhibits angiogenesis needs further investigation.

In the last decade, the link between inflammation and angiogenesis has become recognized. Extensive angiogenesis was observed in chronic inflammation, for example in rheumatoid arthritis and inflammatory bowel disease^30, 31^. It was found that IL-38 reduced the production of proinflammatory cytokines^20, 32^ and raises the question whether IL-38 modulates angiogenic response by modulating proinflammatory reaction. During inflammation, ECs become activated and recruit immune cells from the circulation into the underlying tissue where they play a role in angiogenesis with the up-regulation of growth factors and cytokines. IL-1 and most related family members are primarily inflammatory-associated cytokines and the role of IL-1 molecules in promoting angiogenesis had been studied, such as IL-1α, IL-1β, IL-18, IL-33 and IL-37^7, 17–19^. It was found that IL-1β can mobilize endothelial progenitor cells (EPCs) by regulation of VEGF and VEGFR2 expression on ECs in a VEGF-dependent manner^33^. Supernatant from macrophages of IL-1β knockout mice did not induce inflammatoy or angiogenic responses^34^. We also found that the expression of pro-inflammatory cytokines such as TNF-α, IL-1β and IL-6 were decreased in IL-38-treated mice (Supplementary Figure 2). These results indicate that IL-38 modulates angiogenic response by modulating proinflammatory reaction. In addition, systemic administration with IL-1Ra prevented neovascularization formation in corneas by regulation of VEGF or bFGF^35^. The binding of IL-1Ra and IL-36Ra to their receptor reduces inflammation by blocking the binding of receptor ligands. IL-38 was reported to bind to the IL-1RI because of its highly amino acid homology to the IL-1Ra and IL36-Ra. IL-38 may via binding of IL-1RI inhibit the function of IL-1β and IL-1α to and thus regulates the inflammation-induced angiogenic responses. However, subsequent study indicated that IL-38 binds to the IL-36R not to the IL-1RI, IL-18R and IL-1RAcP^20^. The issues need further study.

The link of IL-38 and Th17 cells was attracted attention recently. Study demonstrated that IL-38 modulates the production of characteristic cytokines of the Th17 response, such as IL-17, IL-22, IL-23, IL-6 and IL-8 by blocking the IL-1R, IL-18R and IL-36R pathways^20^. IL-38 reduced the expression of *C*. *albicans*-induced IL-17 and IL-22 from peripheral blood mononuclear cells by reducing the stimulation of proinflammatory cytokines such as IL-8 in the tissues, similar to IL-36Ra^20, 32^. IL-8 as a known angiogenic factors which promotes angiogenic responses *in vivo* models, has been confirmed by many researchers^36–38^. This might be another mechanism of IL-38 that exerting antiangiogenic function, which needs further study. The identification of IL-38 as antiangiogenic cytokine and its mechanism have important clinical implications. However, the IL-38-related signaling pathway and many others are poorly understood and require further investigation.

## Materials and Methods

### Animals

C57BL/6 mice for oxygen-induced retinopathy model were purchased from the Animal Laboratory of Zhongshan Ophthalmic Center (Guangzhou, China). All animal experiments were approved by the Animal Ethical Committee at Zhongshan Ophthalmic Center, Sun Yat-sen University (Permit Number 2013–084) and were performed in accordance with the guidelines of the Association for Research in Vision and Ophthalmology (ARVO) for the use of animals in research. Animals were kept in a specific pathogen-free facility. Animals care and use were in compliance with institutional guidelines.

### Cells and Culture

Human retinal endothelial cells (HRECs) and Human umbilical vein endothelial cells (HUVECs) were purchased from ScienceCell (Carlsbad, CA) and maintained in ECM according to company’s instructions. Specifically, ECM (ScienCell) supplemented with 5% FBS and endothelial cell growth supplement (ECGS, ScienCell) according to company’s instructions.

### Reagents

Recombinant human IL-38 was purchased from R&D Systems (Minneapolis, MN). Recombinant mouse IL-38 was purchased from Adipogen (AG-40B-0101). Purified Human IgG was purchased from R&D Systems (Minneapolis, MN). For Western blot analysis, antibody against IL-38 was purchased from Abcam (Cambridge, MA). Recombinant human VEGF-A was obtained from R&D Systems (Minneapolis, MN). Endothelial Cell Medium (ECM) were purchased from ScienCell (Carlsbad, CA)

### Mouse Retinal Vascularization

Neonatal mice were intraperitoneally administrated with IL-38 at the concentration of 1 ng/gram bodyweight from P1 to P4. Littermate controls were administrated with the same volume of PBS. The whole retina mounts were isolated at P5 and stained with FITC-conjugated Isolectin B4 (IB4). Retinal vasculature was assessed by fluorescent microscopy and vascular area was analyzed with Image J.

### Mouse Model of Oxygen-induced Retinopathy

Neonatal mice and their nursing mothers were placed in a 75% oxygen supply chamber from postnatal day 7 (P7) to P12. Mice were returned to normal oxygen supply (21%) at P12. Mice were intravitreal administration with 1 μl of IL-38 dissolved in phosphate buffered saline (PBS) at different concentrations (0, 1, or 5 ng/μl) at P12 or intrapertoneal administration with PBS or IL-38 at 1 ng/gram bodyweight at P12, P14 and P16. At P17, the whole retinal mounts were isolated and stained with Isolectin B4 (Sigma-Aldrich, St. Louis, MO). Neovascular region (tufts) and avascular area were analyzed using Image J. Significant differences were determined using Student’s *t* test.

### Real-time quantitative RT-PCR

For IL-38 expression, total RNAs were extracted from normal and OIR mouse retinas, and real-time PCR was performed. For pro-inflammatory cytokines expression under IL-38 administration in OIR model, total RNAs IL-38-treated retinas and control retinas, and real-time PCR was performed. Each reaction contained 12.5 μl of 2 × SYBR green Master Mix, 300 nM oligonucleotide primers (Table 1) synthesized by Invitrogen Biotechnology Co. Ltd, (Shanghai, China), 10 μl of 1 in 10 dilution of the cDNA and water, to a total of 25 μl. The tested mRNA expression was finally determined after correction with GAPDH expression.

**Table 1:** Primer Sequences of mouse for Real-Time RT-PCR.

Gene		Sequence (5′to 3′)	Product Length
IL-38	Sense	GCCTGGCGTGTGTAAAGACA	76
	Antisense	CCCTTGTATAGGTCCTCGATGTT	
TNF-α	Sense	AGACAGAGGCAACCTGACCAC	102
	Antisense	GCACCACCATCAAGGACTCAA	
IL-1β	Sense	GGTAAGTGGTTGCCCATCAGA	88
	Antisense	GTCGCTCAGGGTCACAAGAAA	
IL-6	Sense	GTCACCAGCATCAGTCCCAAG	97
	Antisense	CCCACCAAGAACGATAGTCAA	
GAPDH	Sense	TGAGCAAGAGAGAGGCCCTATC	93
	Antisense	AGGCCCCTCCTGTTATTATG	

### Western Blot

For IL-38 expression, proteins were extracted from normal and OIR mouse retinas at P17. Proteins were detected by antibodies against IL-38 (R&D Systems, Minneapolis, MN). Densitometric quantitation of was performed and analyzed using two-tailed Student’s *t* test.

### Proliferation

HUVECs were cultured in human endothelial-SFM (Invitrogen, Carlsbad, CA). Triplicate 0.2-ml cultures containing 2 × 10^4^ cells were seeded in round-bottom 96-well microtiter plates. The cells were treated with VEGF/IL-38/anti-IL-38/IgG at 37 °C in a CO_2_ incubator for 48 h. MTT solution (10 μl of 5 mg/mL) was added to each well and the cells were further incubated for 4 h at 37 °C. The cells were then resuspended in 100 μl of 0.04 M HCl/isopropanol solution and the incubation was continued for 2 h to solubilize formazan violet crystals in the cells. The absorbance in each well was determined by spectrophotometry at the dual wavelengths of 570 and 630 nm on a microplate reader (Pharmacia, Swedn).

### Migration assays

HUVECs were seeded in a 6-well plate at a density of 1 × 10^6^ cells/well in growth medium until they reached 90% confluence. A scratch was made through each well using a sterile tip. The monolayer was incubated with a migration assay buffer consisting of serum-free medium and VEGF/IL-38/anti-IL-38/IgG. Images were captured at 0 h and 8 h. The area of healing wound was calculated with Image J software^39^.

### Tube formation assay

Tube formation assay was performed as described in detail^40–42^. Briefly, HUVECs were seeded on Matrigel at 40,000 cells/well in 96-well plates with VEGF/IL-38/anti-IL-38/IgG in depleted medium. The cells were cultured 18 h at 37 °C in a 5% CO_2_ incubator. Photographs were taken on an inverted microscope using a 10 × objective. Tube formation in cell culture was quantified by counting the tube-like structures in the gel and data were presented as the number of branches per field ( × 100). In each experiment, 4 fields were calculated simultaneously and 3 independent experiments were performed.

### Statistical Analyses

Data are presented as means ± SEM. Prism software was used for statistical analyses. Significant differences between paired samples were analyzed with two-tailed Student’s *t* test. A value of *P* < 0.05 was considered significant for all analyses.

## Electronic supplementary material

The effect of IL-38 on angiogenesis in a model of oxygen-induced retinopathy
