# Supplementary material for: The Effect of Interleukin 38 on Angiogenesis in a Model of Oxygen-induced Retinopathy
Source: Sci Rep. 2017 Jun 5;7:2756. doi: 10.1038/s41598-017-03079-z (PMC5459839; doi:10.1038/s41598-017-03079-z)
Supplement: Supplementary file 1 — The effect of IL-38 on angiogenesis in a model of oxygen-induced retinopathy [file 41598_2017_3079_MOESM1_ESM.doc]

**SUPPLEMENTARY MATERIALS**

**The Effect of Interleukin 38 on Angiogenesis in a Model of Oxygen-induced Retinopathy**

Jing Zhang, Ruijuan Zhao, Jianping Chen, Jiayi Jin, Ying Yu, Yunzhe Tian, Weihua Li, Wencong Wang, Hongyan Zhou and Shao Bo Su

**Supplementary Figures and Figure Legends**


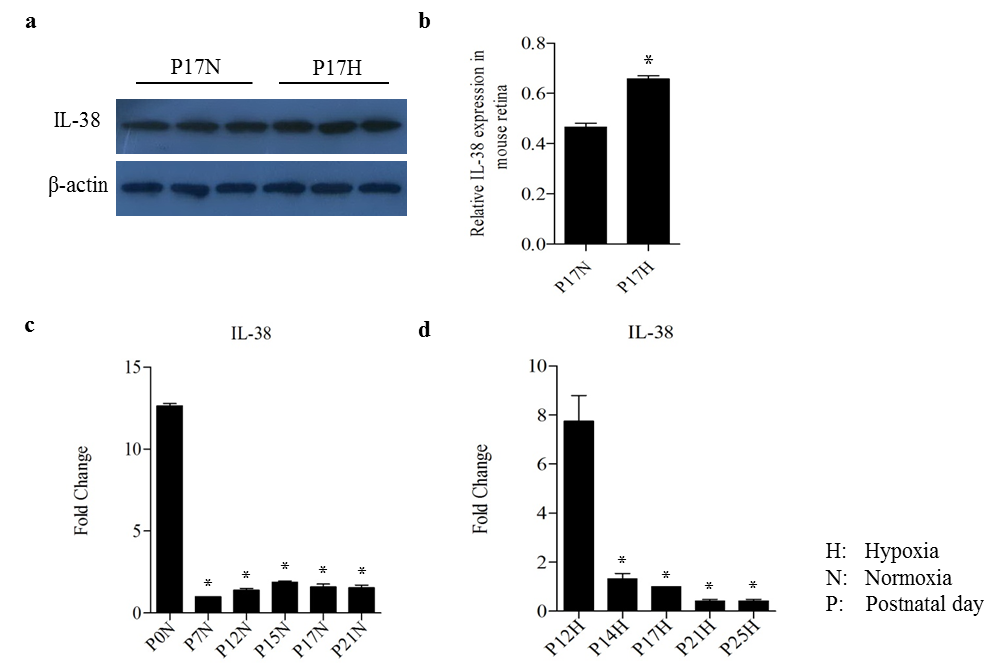


**Supplementary Figure 1.** Interleukin (IL)-38 expression in normal and OIR mouse retina.

**(a)** Western-blot analysis was used to examine IL-38 expression in normal mice (P17) and OIR mice (P17). **(b)** Quantification of (a). **(c)** Real-time PCR analysis was used to examine IL-38 expression at P0, P7, P12, P15, P17 and P21 in normal mice. **(d)** Real-time PCR analysis was used to examine IL-38 expression in OIR mice at P12, P14, P17, P21 and P25. **P*<0.05 Student *t* test.


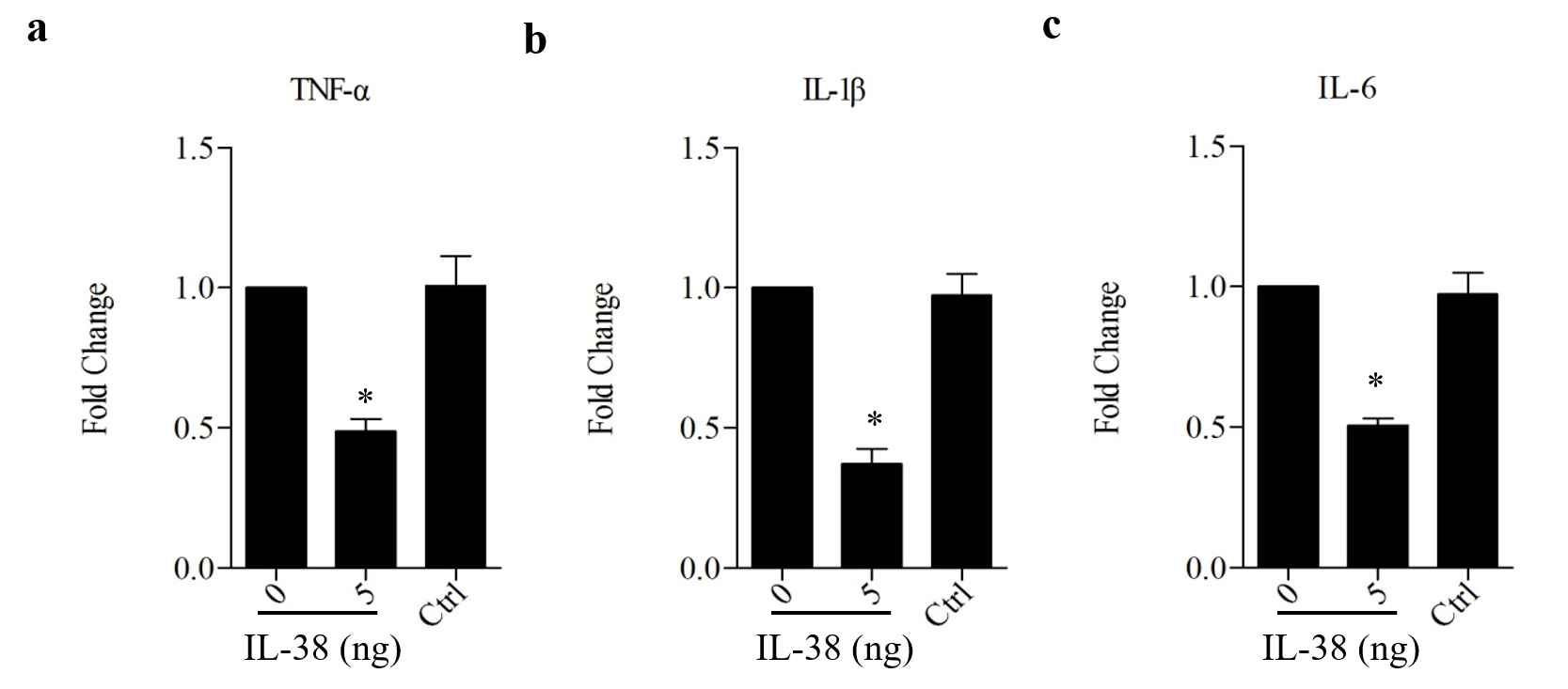


**Supplementary Figure 2.** Expression of pro-inflammatory cytokines by IL-38-treated OIR mouse retinas **(a-c).** The mRNA level for pro-inflammatory cytokines was detected by quantitative PCR and normalized to GAPDH mRNA. **P*<0.05 Student *t* test.


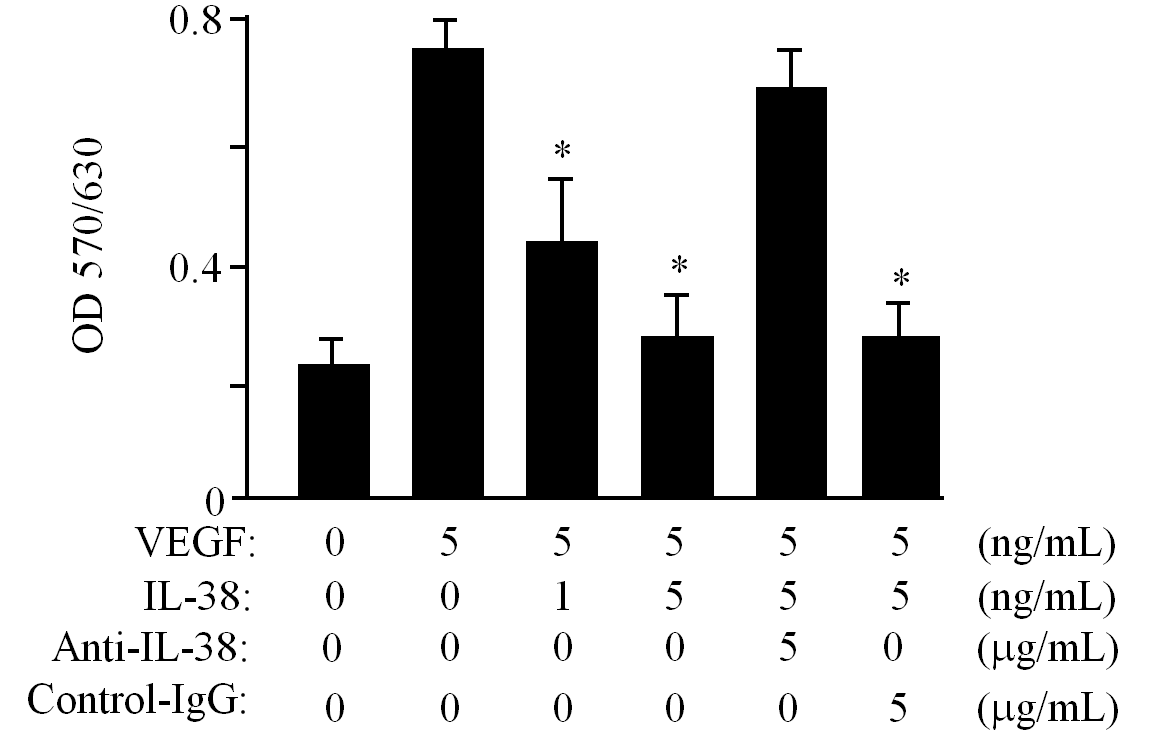


**Supplementary Figure 3. IL-38 attenuates endothelial cell proliferation.** HUVECs (2×10^4^) were cultured with VEGF/IL-38/anti-IL-38/IgG at 37 °C in a CO_2_ incubator and the proliferation was determined by MTT method. Shown are results from one representative experiment of three performed (in triplicates). **P*<0.05 Student *t* test.


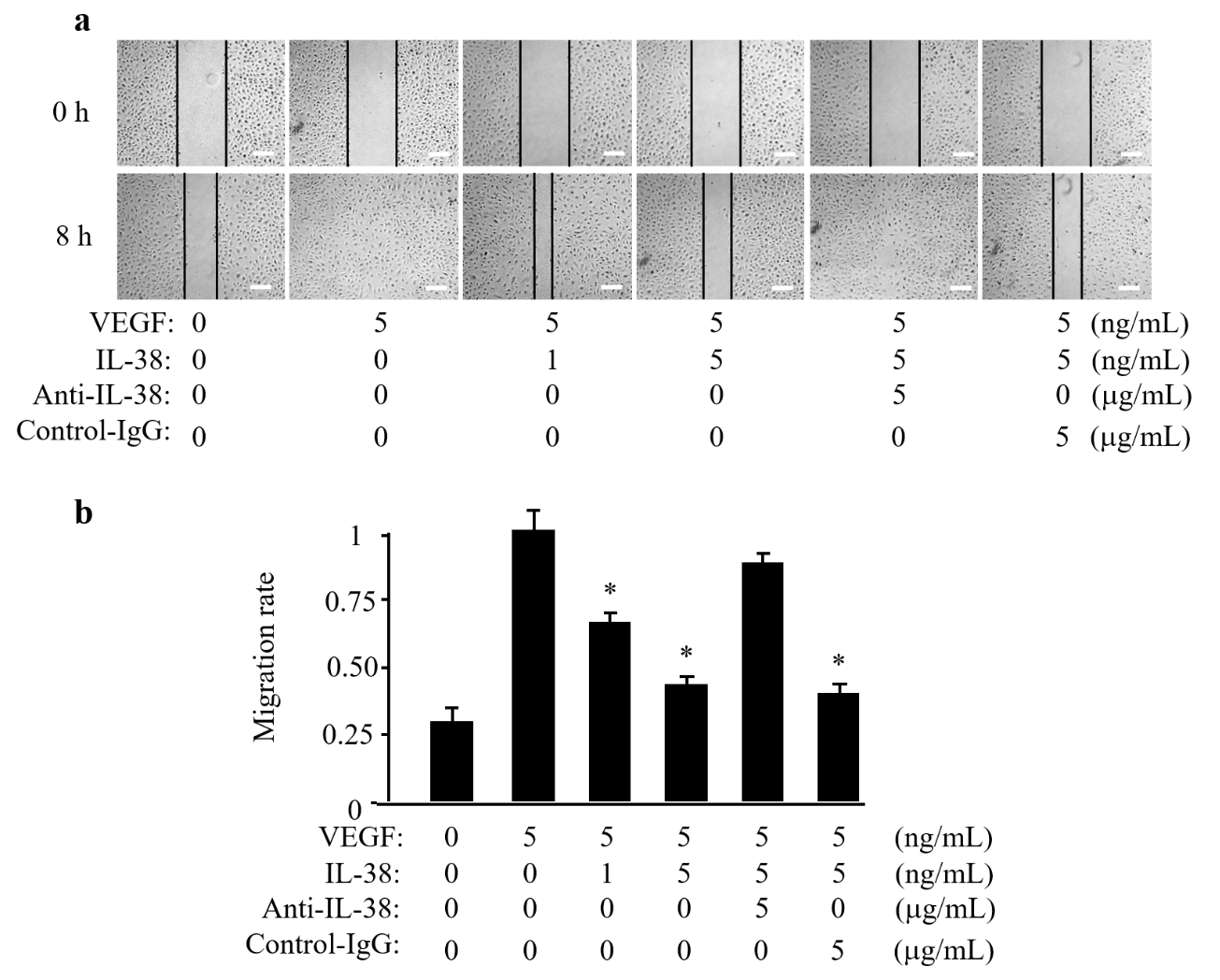


**Supplementary Figure 4. IL-38 reduces endothelial cell migration.** (**a**) The effect of IL-38 on HUVEC migration in scratch wound assay. Cells were treated with indicated concentrations of IL-38. Representative images after 0 and 8 hours after scratch wounding were shown. Scale bars, 100 μm. (**b**) Quantification of IL-38-treated HUVEC migration in monolayer scratch wound assay. **P*<0.05 Student *t* test.


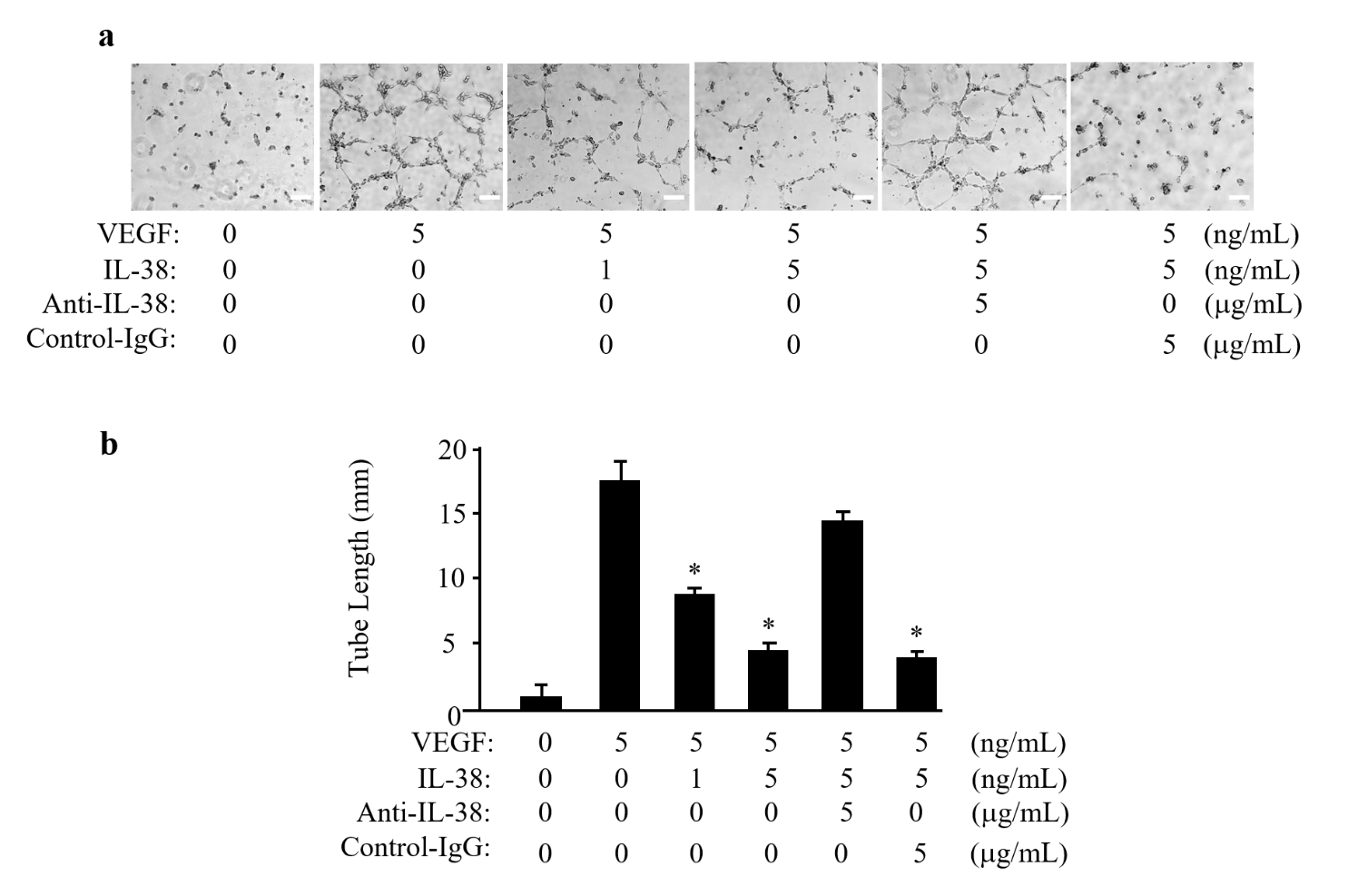


**Supplementary Figure 5. IL-38 reduces endothelial cell tube formation.** (**a**) 40,000 HUVEC cells/well were seeded on Matrigel containing VEGF/IL-38/anti-IL-38/IgG in depleted medium. The cells were cultured for 18 h at 37 ^o^C 5% CO_2_. Tube formation was quantified by counting the tube-like structures in the gel and data were presented as the number of branches per field. Scale bar, 200 μm. (**b**) Total length of tubule structure were quantified. **P*<0.05 Student *t* test.
